# Putative intestinal stem cells

**Published:** 2015

**Authors:** V Pirvulet

**Affiliations:** * Department of Cell Biology and Histology, “Carol Davila” University of Medicine and Pharmacy, Bucharest, Romania

**Keywords:** intestinal stem cells, stem cell ultrastructure, intestinal stem cell niche

## Abstract

A heterogeneous set of intestinal stem cells markers has been described in intestinal glands but the ultrastructural identity of intestinal stem cells remains unknown. By using electron microscopy, this study demonstrated the presence of cells with stem morphology in the intestinal glands of mice of different ages. These putative intestinal stem cells have large, euchromatic, irregular shaped nucleus, large, visible nucleolus, few ER cisternae and mitochondria. Their morphology is distinct from the morphology of any other intestinal gland cell. Stem cells located at the base of intestinal glands undergo mitosis. This study enhances the hypothesis of a gland (crypt) base columnar cell that gives rise to all the intestinal lineages.

## Introduction

The ability to regenerate and replace cells is vital for the viability and homeostasis of most epithelial tissues, including the intestinal tract. Cellular regeneration typically depends on stem cells: primitive and relatively unspecialized cells in fetal and adult tissues that have properties of self-renewal, clonogenicity and multipotency [**1**].

The presence of adult stem-like cells in the gastrointestinal tract was first postulated by Charles LeBlond 60 years ago [**2**], before they were recognized in other organs. Adult stem cells, such as intestinal tissue stem cells, lack cell specific patterns of expression but give rise to the so-called progenitor cells. These, in turn, produce cellular descendants that have a more restricted lineage potential [**3**]. There is an ongoing debate about how many intermediate cell entities, such as progenitor cells, exist [**4**].

Stem cells in the intestine are located in specific sites within the epithelium, adjacent to areas of rapid proliferation and high cell turnover. Proliferation occurs at the base of intestinal crypts in the small intestine; most of the cells migrate up from the crypts to the villi, while some of the cells migrate below the stem cells to form Paneth cells. A few enteroendocrine, mucus and columnar cells might also migrate downward from the common origin into cell positions 1–4 [**5**].

In 2007, a single marker, LGR5, a leucine-rich orphan G protein-coupled receptor, was identified in lineage-tracing studies to specifically label stem cells in the mouse small intestine, such as the crypt base columnar cells between the Paneth cells [**6**]. This research has reactivated the debate about the location of intestinal stem cells. Some LGR5-positive cells seem to be multipotent and are able to form all mature intestinal epithelial cells. They seem to undergo self-renewal, to persist for several months and to be resistant to irradiation. Thus, these rapidly proliferating cells with intestinal stem cell characteristics have challenged the previously held belief that all adult stem cells are generally quiescent or slowly cycling [**7**]. 

In 2009, lineage-tracing studies of adult prominin-1 (also called CD133; a pentaspan transmembrane glycoprotein that localizes to membrane protrusions) showed that some prominin-1-positive cells are located at the base of crypts in the small intestine, co-express LGR5 and can generate the entire intestinal epithelium, and therefore seem to be small intestinal stem cells as well [**8**,**9**].

**Table 1:** Intestinal tissue stem cell markers

Marker	Characteristics of cells
LGR5	Active cycling crypt base columnar cells that give rise to all intestinal lineages (lineage tracing) [**6**]
Prominin-1	Active cycling crypt base columnar cells that give rise to all intestinal lineages (lineage tracing), overlaps with LGR5 [**8**-**10**]
BMI1	Quiescent cells around position 4+ that give rise to all intestinal lineages (lineage tracing) [**11**]
DCLK1	Expression around position 4+ (no lineage tracing) [**12**,**13**]
CCK-BR	Probably present on, but not specific for colonic stem cells or progenitor cells [**14**]
Label retaining (BrdU)	Quiescent cells at position 4+ [**15**]

**Fig. 1:**
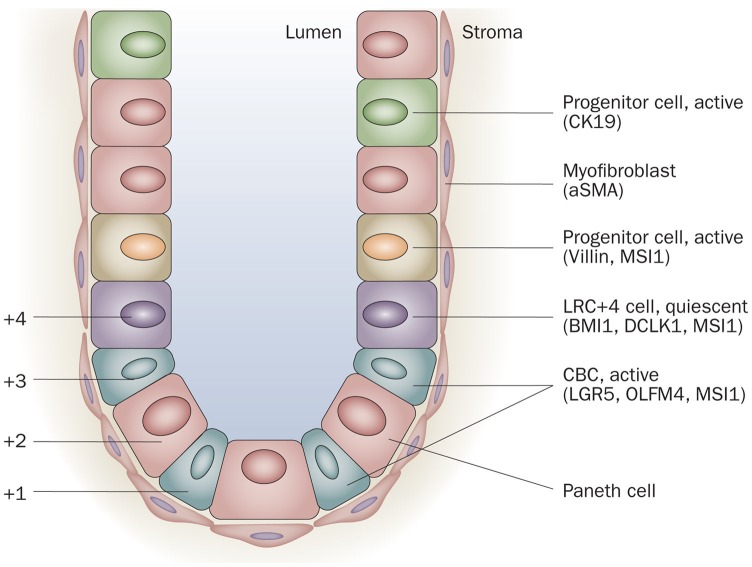
Schematic illustration of the location of putative intestinal stem cells and/ or progenitor cells and their markers in the crypt of the intestine. Quiescent stem cells may be located at position +4, the more active stem cells (crypt base columnar cells [CBCs]) are located anywhere from position +1 to +4 scattered between the Paneth cells. The intestinal glands are surrounded by stromal cells (niche cells), such as myofibroblasts. Quante M, Wang TC. Physiology. Bethesda. 2008; 23, 350–359

This paper tried to identify the putative intestinal stem cells in their stem cell niche, intestinal cells progenitors and their morphology in different developmental stages, by electron microscopy, from two weeks to adulthood in mice, in a comparative study with the literature data.

The features of putative intestinal stem cell are not yet known and their ultrastructural phenotype(s) should be of great interest for their characterization.

## Materials and Methods

Transmission electron microscopy

Small tissue fragments (about 1mm3) from mouse intestine were fixed in 4% glutaraldehyde solution (in 0.1M cacodylate buffer), prepared fresh for 4 h at 4°C. After a brief wash of the samples in 0.1M sodium cacodylate the solution was followed by a step of postfixation at room temperature for 60 minutes in a mixture of 1% potassium ferrocyanide and 1% osmium tetroxide in 0.05 M sodium cacodylate buffer (pH 7.4).

Samples were then dehydrated in solutions with increasing ethanol concentrations. After impregnation of propylene, the tissue was immersed overnight in a mixture of propylene oxide and resin Epon 812 and Epon included in the section has been made ultrafine (50 nm), by using ultramicrotome MT 7000 (Research Manufacturing Company, Inc., Tucson, AZ, USA), after which they were mounted on copper grids and contrasted with uranyl acetate and Reynolds’ lead citrate.

Digital images were taken with MegaView III CCD camera, operated by iTEM- the SIS software (Olympus Soft Imaging System GmbH, Germany) and transmission electron microscope mounted Morgagni 286 TEM (FEI Company, Eindhoven, The Netherlands) at 60 KV.

## Results

While using electron microscopy and exclusion criteria, it was found that some intestinal epithelial cells presented ultrastructural features of stem cells. These putative intestinal stem cells have been found in specific areas of the epithelium, adjacent to the rapidly proliferating area.

**Fig. 2:**
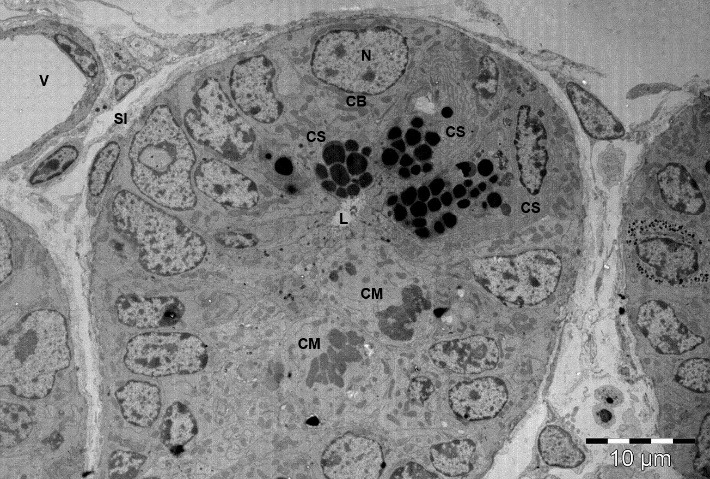
Transmission electron microscopy image of the basal area of the Lieberkuhn gland of the small intestine (two-weeks-old mouse). Cells with morphology of stem cells were observed at the base of Lieberkuhn gland of the intestinal mucosa along with secretory cells and two cells in mitosis in a higher floor. This image shows putative intestinal stem cells in intestinal stem cell niche. L-intestinal lumen; CM-cells in mitosis; N- nucleus; CB-basal cell; CS-secreting cell; SI-interstitial space; V-blood vessel

Transmission electron microscopy (**Fig. 2**) showed a cross section through a Lieberkuhn gland from small intestine of a two-week old mice, in which two dividing cells could be seen near the lumen, considered according to literature precursor cells and at the basis of the gland, besides Paneth cells, cells with ultrastructural appearance like young cells: large nucleus (core report/ cytoplasm above par), euchromatic, visible nucleolus and cytoplasm with few organelles: the mitochondria and endoplasmic reticulum few tanks, considered to be stem cell, corresponding to literature data as gland position (**Fig. 1**).

Cells presented in this paper as putative stem cells or progenitor cells have been found to undergo mitosis (**Fig. 2**).

**Fig. 3:**
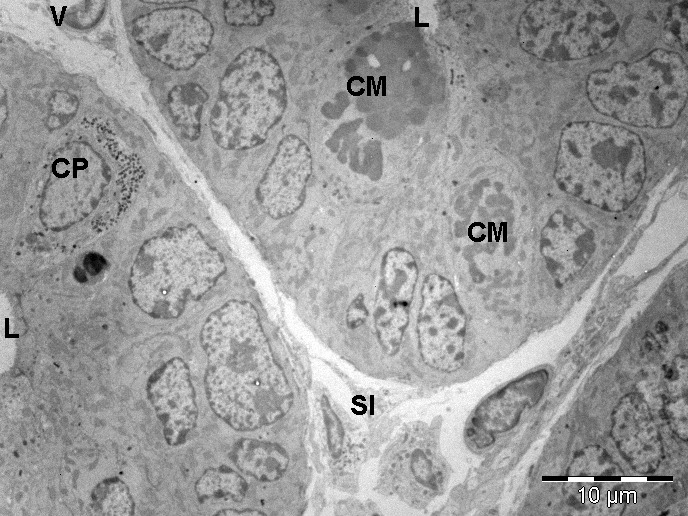
Transmission electron microscopy image of basal areas of Lieberkuhn glands of the small intestine (two-weeks-old mouse). Cells with morphology of stem cells were observed at the base of Lieberkuhn gland of the intestinal mucosa along with a Paneth cell and two cells in mitosis in different floors. L-intestinal lumen; CM-cells in mitosis; N-nucleus; CP-Paneth cell; SI-interstitial space; V-blood vessel

Putative intestinal stem cells or cells with stem cell morphology or basal cells are cells with large, euchromatic, irregular shaped nucleus, large nucleolus, few endoplasmic reticulum cisternae and mitochondria.

**Fig. 4:**
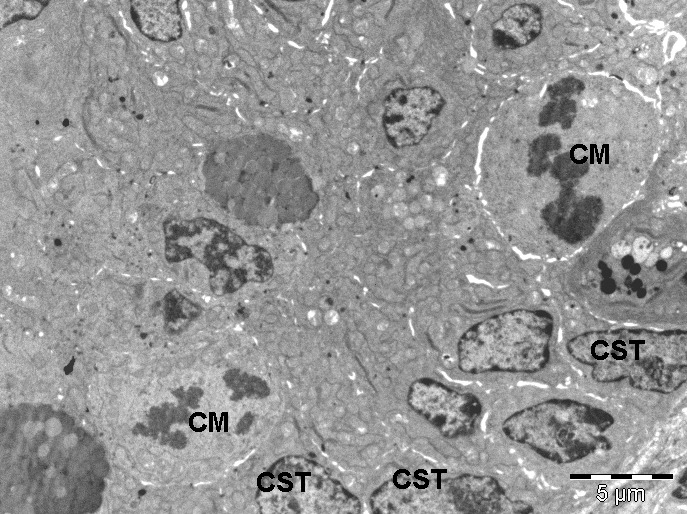
Transmission electron microscopy image showing small intestine epithelium (three-months-old mouse). Cells with morphology of stem cells were observed between the other epithelial cells. CM-cells in mitosis; CS-cells with morphology of stem cells

**Fig. 5:**
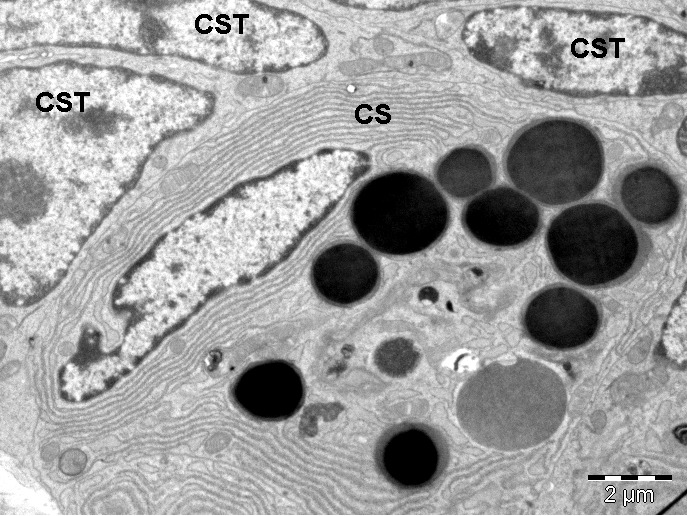
Transmission electron microscopy image showing small intestine epithelium (one-year-old mouse). Cells with morphology of stem cells were observed between the other gland cells. CST- cells with morphology of stem cells, CS-secreting cell

## Discussion

Tissue-restricted stem cells are generally difficult to identify morphologically and are not easily distinguished from other epithelial cells by any consistent set of markers, except for perhaps their ability to divide and self renew [**16**,**17**]. Tissue stem cells or progenitor cells are thought to reside within a “niche”—an area with extracellular substrates that provide an optimal microenvironment for normal differentiation. Progenitor cells divide quickly and are responsible for the bulk of cell division, but seem to have a limited lifespan and are replaced periodically by descendents of the true stem cell [**18**].

Studies in transmission electron microscopy have shown that in different sections, the cell with the particular morphology does not resemble any other intestinal epithelial cell morphology, but are young cell-like cell morphology, undifferentiated or stem cells.

The location of these cells was consistent with the location of the stem cell markers in literature data.

In comparison, a decrease in mitosis was observed to be present in the intestinal epithelium with age, most present on the pictures taken from the intestine of two-weeks-old mice.

The next step would be to identify the stem cell markers on electron microscopy studies along with the stem cell microenvironment or niche and with the signals that regulate the behavior of these stem cells.

**Acknowledgements**

The author is grateful to Mihaela Gherghiceanu, MD, “V. Babes” National Institute of Pathology for expert assistance in electron microscopy.

## References

[1] Reya T, Morrison SJ, Clarke MF, Weissman IL (2001). Stem cells, cancer, and cancer stem cells. Nature.

[2] Leblond CP, Stevens CE, Bogoroch R (1948). Histological localization of newly-formed deoxyribonucleic acid. Science.

[3] Rossi DJ, Jamieson CH, Weissman IL (2008). Stems cells and the pathways to aging and cancer. Cell.

[4] Chuong CM, Widelitz RB (2009). The river of stem cells. Cell Stem Cell.

[5] Bjerknes M, Cheng H (1981). The stem-cell zone of the small intestinal epithelium. III Evidence from columnar, enteroendocrine, and mucous cells in the adult mouse. Am J Anat.

[6] Barker N (2007). Identification of stem cells in small intestine and colon by marker gene Lgr5. Nature.

[7] Barker N, Clevers H (2007). Tracking down the stem cells of the intestine: strategies to identify adult stem cells. Gastroenterology.

[8] Zhu L (2009). Prominin 1 marks intestinal stem cells that are susceptible to neoplastic transformation. Nature.

[9] Snippert HJ (2009). Prominin-1/ CD133 marks stem cells and early progenitors in mouse small intestine. Gastroenterology.

[10] Vermeulen L (2008). Single-cell cloning of colon cancer stem cells reveals a multi-lineage differentiation capacity. Proc Natl Acad Sci USA.

[11] Sangiorgi E, Capecchi MR (2008). Bmi1 is expressed in vivo in intestinal stem cells. Nat Genet.

[12] Giannakis M (2006). Molecular properties of adult mouse gastric and intestinal epithelial progenitors in their niches. J Biol Chem.

[13] May R (2008). Identification of a novel putative gastrointestinal stem cell and adenoma stem cell marker, doublecortin and CaM kinase-like-1, following radiation injury and in adenomatous polyposis coli/ multiple intestinal neoplasia mice. Stem Cells.

[14] Jin G (2009). Inactivating cholecystokinin-2 receptor inhibits progastrin-dependent colonic crypt fission, proliferation, and colorectal cancer in mice. J Clin Invest.

[15] Potten CS, Kovacs L, Hamilton E (1974). Continuous labelling studies on mouse skin and intestine. Cell Tissue Kinet.

[16] Booth D, Haley JD, Bruskin AM, Potten CS (2000). Transforming growth factor-B3 protects murine small intestinal crypt stem cells and animal survival after irradiation, possibly by reducing stem-cell cycling. Int J Cancer.

[17] Booth C, Potten CS (2000). Gut instincts: thoughts on intestinal epithelial stem cells. J Clin Invest.

[18] Brittan M (2002). Bone marrow derivation of pericryptal myofibroblasts in the mouse and human small intestine and colon. Gut.

